# GPs' perceptions of digital technology for behavior change interventions for community-dwelling older adults: a cross-sectional study

**DOI:** 10.3389/fpubh.2026.1849754

**Published:** 2026-07-15

**Authors:** Yixuan Zeng, Shuai Fang, Yan Liang, Yan Hu

**Affiliations:** 1 School of Public Health, Fudan University, Shanghai, China; 2 Institute of Sociology, Shanghai Academy of Social Sciences, Shanghai, China; 3 School of Nursing, Fudan University, Shanghai, China

**Keywords:** behavior, behavioral sciences, general practice, primary health care, technology

## Abstract

**Introduction:**

Digital technologies have shown great potential in supporting behavior change interventions for older adults. However, general practitioners' (GPs) perceptions regarding using digital technologies to implement behavior change techniques (BCTs) in primary care settings remain unclear, especially in China. This cross-sectional study explored GPs' perceptions of such digital support and concurrent associations between perceived necessity, self-reported competence, and perceived usefulness.

**Methods:**

A convenience sample of 540 GPs from three Shanghai districts completed an online survey between December 1, 2023 and December 10, 2023. Separate clustering-robust logistic regression models were fitted for each of 16 BCT groups (with sociodemographic and occupational variables as confounders); given only three district-level clusters, inferential results are interpreted as exploratory rather than definitive.

**Results:**

GPs generally recognized BCT necessity but reported relatively low competence. Perceived necessity and self-reported competence were positively associated with perceived usefulness across all 16 BCTs (all FDR-adjusted *p* < 0.001). Descriptively, necessity's odds ratios were numerically higher in 15 BCTs, but this was a non-inferential descriptive pattern. Perceptions varied descriptively by BCT type; self-reported competence showed a wider endorsement spread than perceived necessity.

**Discussion:**

This exploratory study provides initial insights into GPs' perceptions in selected Shanghai community health service settings, highlighting key cognitive associations and a necessity-competence discrepancy. Digital technologies may support BCT delivery, but findings are tentative due to study limitations including single-item dichotomous measures, few district-level clusters, and within-respondent dependence across 16 BCT judgments. Rigorous future research is needed to validate patterns and inform initiatives.

## 1 Introduction

Behavior change techniques (BCTs) refer to the observable, reproducible, and irreducible components of interventions designed for behavior change (1). As the understanding of BCTs continues to deepen, their application in various fields has gradually expanded, especially in health. From physical to psychological, BCTs are widely used to promote health. For example, BCTs increase older adults' self-efficacy and physical activity behavior (2) and improve mental health service utilization for males (3). As the aging trend in China continues, the proportion of people aged 60 and above will exceed 20% of the total population (4). The health issues of the vast number of older adults living in the community and related BCTs are becoming a growing concern for policymakers and researchers. In China, general practitioners (GPs), often referred to as “family doctors” serve as the gatekeepers of the healthcare system under the hierarchical diagnosis and treatment framework. Since the rollout of the family doctor contract service policy in 2016, GPs have been mandated to provide continuous, comprehensive care—including health management and behavior change counseling—for community-dwelling older adults. However, their role differs from that in many Western contexts: Chinese GPs typically face heavy administrative burdens, high patient volumes, and limited consultation time, which may constrain their capacity to deliver individualized BCTs.

Meanwhile, as the older adults' demand for health services increases, and with the improvement of China's hierarchical diagnosis and treatment system (5), the primary medical and health team focusing on GPs has gradually assumed this responsibility. GPs are considered appropriate and necessary healthcare personnel from whom patients can obtain advice on behavior change (6). However, the health management of older people is characterized by long-term complexity; in addition to the increasing base of older people, the pressure on GPs to deliver health services is only increasing. Therefore, finding a new solution or pathway to help GPs effectively manage older people and assist them with BCTs is necessary. Digital technologies may be one of these solutions, as the work of GPs is increasingly characterized by digitalization (7), from long-term adoption of digital tools to teleconsultation and AI-based medical decision support systems (8).

Over the past few years, devices and programs that use digital technologies to promote or support behavior change have become increasingly common for patient diagnosis and treatment, self-management of chronic diseases, and primary prevention (9). For example, in Australia, it has been proven that a digitally delivered professional behavior change intervention was comparable to a postal intervention and had superior efficacy for referral services (10). Emma at el. (11) identified BCTs that patients preferred for each health behavior targeted in eHealth-based cardiac rehabilitation so that they can further develop and apply interventions for specific patients. Behavior change wheel theory was used on an interactive digital health outreach platform to reveal potential mechanisms for improving medication adherence (12) and a digital self-management program for people with chronic kidney disease (13). Diseases like type 2 diabetes, hypertension, obesity, and cardiovascular diseases are closely linked to modifiable health behaviors. BCTs have demonstrated effectiveness in improving outcomes for these conditions by promoting behavioral changes such as healthier eating (14), regular physical activity (15), and medication adherence.

Many studies focus on using digital technology to intervene in older adults' behavior change by GPs, such as digital intervention to support antidepressant discontinuation, video or online consultation, digital coaching using behavioral change techniques for weight loss, digital health profile for health check, and behavior-change counseling (16–21). However, research on GPs' perceptions of using digital technology-supported behavior change interventions for community-dwelling older adults is lacking. What's more, perceived usefulness is defined as “the degree to which a person believes that using a particular system would enhance his or her job performance.” This means users adopt an application to the extent that they believe it improves job performance (22). In the Technology Acceptance Model within the information systems domain, perceived usefulness has been regarded as a key factor in technology acceptance (23). A study on medication adherence apps found that patients' acceptance of these apps was higher when they believed the technology could help them better manage their medication use (24). Thus, investigating GPs' perceived usefulness of digital technology in conducting BCTs can assess their eagerness to use a digital tool (25).

The influencing factors of perceived usefulness have been discussed widely. Several classic theories in health informatics and motivational psychology—including the Technology Acceptance Model (TAM), expectancy-value theory, and self-determination theory (26–28)—have identified core cognitive constructs relevant to individuals' acceptance of new technologies and behavioral engagement. Drawing on TAM, expectancy-value theory, and self-determination theory as exploratory frameworks—not as directly tested confirmatory models—we examine whether patterns consistent with these theories emerge in an underexplored context. GPs' perceived necessity of a BCT may be aligned with the “expectancy” and “value” components of motivation, while self-reported competence may relate to self-determination theory's concept of perceived competence. Perceived usefulness, drawn from TAM, is examined as an indicator associated with technology acceptance. However, core constructs such as perceived ease of use, behavioral intention, subjective norms, autonomy, and actual digital technology adoption were not directly measured. By examining three coarse exploratory indicators simultaneously across 16 BCTs, this study explores whether motivational and technology-acceptance-related patterns may be consistent with these frameworks in selected Shanghai primary care geriatric management settings. Perceived necessity and self-reported competence are examined as constructs potentially associated with the perceived usefulness of digital technologies in supporting BCTs. Perceived necessity refers to the extent to which GPs believe that a particular BCT is essential for managing the health of older adults. Self-reported competence refers to the extent to which GPs feel they have the skills to implement a specific BCT in general clinical practice (not specifically via digital technology). While theoretical models suggest necessity and competence may precede usefulness in the formation of technology acceptance perceptions, the cross-sectional nature of this study limits us to examining the concurrent associations among these constructs. Relevant evidence has shown that healthcare providers' self-assessment of their professional abilities is closely linked to their acceptance of technological tools in clinical practice (29), which further supports the rationality of exploring the correlations among these three constructs in this study.

Patient behavior is one of the top services that GPs prioritize (30). Previous studies proved that GPs are familiar with BCTs and are ready to use them (31), and GPs are fundamental to the success of the tool's use and achieving change in the target behavior (32). The confidence and skills GPs have in using digital technologies bring new complexities and concerns (33). Their perception gives insights for the future development of digital technologies, which promote behavior change by implementing new health behaviors (34). However, no studies have investigated the perception of Chinese GPs using digital technologies to assist in implementing BCTs to manage older adults' health. Thus, the study aims to explore GPs' perceptions of the use of digital technologies in specific BCTs in China, and the associations between perceived necessity, self-reported competence, and perceived usefulness. It should be made explicit that this study focused on GPs' views about using technology in primary care, not on older adults' behavior, and no patient-level inferences were drawn. The present study, however, does not examine the actual implementation of BCT interventions, nor their effects on behavior change or patient health outcomes; instead, it focuses on GPs perceptions of using digital technologies to deliver these BCTs in primary care settings for community-dwelling older adults.

## 2 Method

### 2.1 Study design

This is a cross-sectional study. A survey was undertaken to explore GPs' perceptions of digital technologies in specific BCTs in China and identify the relationship between perceived necessity, self-reported competence, and perceived usefulness. Burns et al. (35)'s guidelines guided the survey development. Survey items were developed through literature reviews, and we involved five content experts (including two community health center managers and three experts from health policy and primary care) to review the draft questionnaire for relevance, clarity, meaningfulness and completeness. Both item-level (I-CVI) and scale-level (S-CVI) content-validity indices were calculated. To verify that the wording would be interpreted as intended, we subsequently conducted interview-based appraisals with colleagues similar to target respondents, assessing the appropriateness of every item. The study's reporting follows the STROBE Checklist for reporting cross-sectional studies.

The survey was developed in English based on the BCT Taxonomy (v1) and the Technology Acceptance Model literature, then translated into simplified Chinese by two bilingual researchers (native Mandarin speakers with advanced English proficiency). A third independent bilingual expert back-translated the Chinese version into English. The research team compared the back-translation with the original English version and resolved discrepancies through consensus. The final Chinese questionnaire was piloted with 10 GPs from a non-participating community health center to ensure linguistic clarity and cultural appropriateness. All participants completed the survey in Chinese; no language barriers were reported.

### 2.2 Setting and participants

The study involved GPs in community health services centers in Shanghai, China's Changning, Huangpu, and Jinshan districts. Using convenience sampling, we targeted GPs working in community health service centers across three Shanghai districts (Changning, Huangpu, and Jinshan), selected to reflect both urban-core and suburban settings. Through district health commission networks, email, and WeChat, we distributed an anonymous web-based questionnaire between December 1, 2023 and December 10, 2023. A total of 784 GPs were approached via professional networks; 540 completed the survey (response rate 68.9%). The inclusion criteria were as follows: (1) working as a GP in community health service centers in the Changning, Huangpu, or Jinshan district of Shanghai, (2) able to understand the research purpose and voluntarily participate in the research. GPs who were not actively practicing during the study period (e.g., on long-term leave) were excluded.

### 2.3 Ethical considerations

This study received approval from the ethics review committee of the school of nursing, Fudan University (IRB#2023-4-7). The online questionnaire and informed consent forms were provided to participants via a link. Only when the participant clicked “agree to participate” could they enter the questionnaire to ensure that all participation was voluntary. Participants could withdraw at any time by discontinuing the survey without submitting; only submitted questionnaires were included in the analysis. To minimize missing data, all items were required and the platform permitted submission only after every item had been answered, consistent with the approved study protocol. The questionnaire itself did not contain any personally identifiable information, and only occupational characteristics relevant to the research topic were collected. Research data are only used for statistical analysis and results presentation of this study, and will not be disclosed or shared with any third party. There was no financial or material incentive for taking part in the survey.

### 2.4 Measures

The questionnaire was constructed based on previous literature regarding the use of technologies in healthcare practice and BCTs (36, 37). Following the prior study (1), 93 BCTs were clustered into 16 groups for the following reasons: (1) The selected BCTs are those that have strong evidence supporting their effectiveness in behavior change mechanisms (38). They are specifically targeted at physical activity among the older adults and are the most effective in enhancing self-efficacy and promoting physical activity (2). (2) In the prior intervention research, a core set of effective BCTs was actually used rather than attempting to cover all possibilities (39). (3) Considering the working context of GPs in China, such as high patient load, heavy administrative tasks, and short consultation time, and based on the specific research context and population (Chinese older adults and GPs) (40), a more streamlined and focused subset of BCTs was chosen to ensure the feasibility of the investigation.

#### 2.4.1 . Necessity, competence of BCTs, and usefulness of digital technology-supported BCTs

We investigated GPs' perceptions of using digital technology-supported behavior change interventions for community-dwelling older adults, including necessity and self-reported competence of BCTs, and perceived usefulness of digital technology-supported BCTs. 16 BCTs (Supplementary Table S1) were chosen based on the hierarchical classification system developed by Michie et al. (1). These BCTs were selected due to their relevance and applicability in primary care settings for managing the health of older adults. The selection aimed to cover a broad range of techniques that GPs might use or consider using in their practice.

For each BCT, we used three single-item dichotomous measures. These items serve as coarse exploratory indicators of perceived necessity, self-reported competence, and perceived usefulness, rather than validated multi-item scales of the underlying theoretical constructs. The exact wording was: a) Perceived necessity: “Please rate based on your perceptions toward the necessity of the BCT in your practice” (Yes/No). b) Self-reported competence: “Do you think you have the ability to conduct the BCT?” (Yes/No). This item refers to the GP's perceived ability to deliver the BCT in general clinical practice, not specifically competence in using digital technology to deliver the BCT. c) Perceived usefulness: “Do you think digital technologies can assist you in conducting the BCT (e.g., improve your intervention, make your intervention more effective or easier)”? (Yes/No). Given that the study involved assessing 16 BCTs, we adopted single-item dichotomous measures to minimize the response burden on GPs and ensure a high response rate and data quality. This approach is consistent with prior studies that used single-item measures (22, 41) and a binary variable (41). Based on the 16 BCTs and three items matrix, 48 questions were generated. If the general practitioner answered “yes,” it was encoded as “1”; otherwise, it was encoded as “0.” The full questionnaire is provided in Supplementary Material A.

#### 2.4.2 . Sociodemographic characteristics

Sociodemographic characteristics included sex, age, educational background, professional title, and years of work.

### 2.5 Statistical analysis

First, descriptive statistics were presented as frequencies and percentages for categorical variables. Second, following precedent from cross-sectional surveys of primary care practitioners' technology-related perceptions (36, 37), we analyzed associations separately for each BCT group. To examine the associations between perceived necessity, self-reported competence, and perceived usefulness for each of the 16 BCTs separately, 16 clustering-robust logistic regression models were constructed. For each BCT, perceived usefulness served as the dependent variable, with perceived necessity and self-reported competence as core independent variables. Sociodemographic characteristics (sex, age, educational background, professional title, and years of work) were included as confounders. Clustering at the district level was addressed by estimating cluster-robust standard errors using the sandwich package (42), which accounts for the non-independence of observations within the same district. Because the study included only three district-level clusters, conventional cluster-robust standard errors have limited reliability and the resulting *p*-values and confidence intervals should not be treated as definitive (42). We therefore interpret inferential results primarily as indicators of consistent association direction across BCTs rather than as precise estimates of district-level population parameters.

We explored a unified mixed-effects logistic regression on long-format data, with GP-level random intercepts and BCT fixed effects to account for within-GP dependence and BCT-specific baseline differences across the 16 repeated judgments per GP. This specification did not include BCT-specific interaction terms and therefore was not intended to provide formal tests of differential association strength across BCTs. In our analysis pipeline, this unified model did not yield stable parameter estimates under the fitted specification, and we were unable to obtain reliable unified-model inference for within-GP dependence in this dataset. We therefore fitted separate per-BCT clustering-robust logistic models as the most stable and transparent approach for this exploratory analysis. Accordingly, all cross-BCT comparisons in this manuscript are strictly descriptive and should not be interpreted as formal tests of differential association strength across BCTs.

Missing data: The online questionnaire required responses to all items before submission; incomplete questionnaires could not be submitted. Accordingly, among the 540 completed questionnaires, no missing data occurred on BCT perception items or sociodemographic variables.

Benjamini-Hochberg false discovery rate (FDR) correction was applied to adjust for multiple testing across the 16 BCTs (32 total tests for necessity and competence) (43).

Model diagnostics were conducted to assess model stability. To verify that predictor-outcome combinations did not exhibit complete or quasi-separation, we examined cell frequencies for all necessity × competence × usefulness combinations within each BCT. All combinations contained both 0 and 1 responses (minimum cell count: 2 observations), indicating that complete separation was not observed; however, several models showed very narrow confidence intervals, consistent with near-quasi-separation or scale compression under dichotomous measurement.

Given the single-item dichotomous measurement approach, we also examined whether inflated OR magnitudes might reflect scale compression rather than large substantive effects. Penalized logistic regression could be applied in separate per-BCT models where needed; an equivalent penalized unified mixed-effects specification was not pursued because the base unified model did not yield stable estimates in our analysis pipeline. The Hosmer-Lemeshow test was also performed to assess the goodness-of-fit of the models.

Sensitivity analyses were conducted in two layers. First, on the pooled sample (all 540 GPs across districts), we compared standard logistic regression (without cluster adjustment) with clustering-robust logistic regression to assess whether association directions were affected by district-level cluster adjustment (Supplementary Table S2). Second, as a district-stratified descriptive sensitivity check, we compared endorsement rates for perceived necessity, self-reported competence, and perceived usefulness across Changning, Huangpu, and Jinshan districts (Supplementary Table S3) to examine whether descriptive patterns (e.g., necessity endorsement exceeding competence) were broadly consistent across districts. This district-stratified comparison is descriptive only and does not constitute formal inference about between-district differences; it is distinct from the pooled-sample comparison in Supplementary Table S2. Neither sensitivity analysis overcomes the fundamental limitation of cluster-robust inference with only three district-level clusters. Odds ratios (ORs) and their 95% CIs were reported to quantify association strength. Statistical significance was defined as an adjusted *P*-value < 0.05. Data visualization was also used for odds ratios with 95% confidence intervals. R (version 4.3.2) was used for all analyses.

## 3 Results

### 3.1 Sample characteristics

Table 1 shows the sociodemographic characteristics of GPs. 784 GPs were approached; 540 completed the survey (68.9% response rate). The majority of the GPs were female (378/540, 70.0%), and nearly half were aged 40–49 (247/540, 45.7%), followed by those aged 30–39 (185/540, 34.3%). Regarding educational background, 80.0% (432/540) held a bachelor's degree, while 12.0% (65/540) had a master's degree or above. Regarding professional titles, 74.1% (400/540) had intermediate or lower titles. Regarding work experience, 45.2% (244/540) had worked for over 20 years.

**Table 1:** Descriptive analysis of sociodemographic characteristics (*N* = 540).

Characteristic	Category	Total, *n* (%)
Sex	Female	378 (70.0)
	Male	162 (30.0)
Age (years)	Under 30	19 (3.5)
	30–39	185 (34.3)
	40–49	247 (45.7)
	Over 50	89 (16.5)
Educational background	Associate degree and below	43 (8.0)
	Bachelor degree	432 (80.0)
	Master's degree and above	65 (12.0)
Professional title	Intermediate title and below	400 (74.1)
	Senior title	140 (25.9)
Years of work (years)	0–5	30 (5.6)
	6–10	96 (17.8)
	11–15	99 (18.3)
	16–20	71 (13.1)
	>20	244 (45.2)

### 3.2 Descriptive endorsement rates for perceived necessity, self-reported competence, and perceived usefulness across 16 BCT groups

Before examining multivariable associations, we summarized GPs' binary responses for each of the 16 BCT groups. The proportion endorsing perceived necessity ranged from 72.0% (Associations) to 88.0% (Shaping knowledge); self-reported competence ranged from 57.6% (Associations) to 80.4% (Shaping knowledge); and perceived usefulness of digital technology support ranged from 64.8% (Comparison of outcomes) to 80.0% (Goals and planning) (Supplementary Table S4). For all 16 BCT groups, the endorsement rate for perceived necessity exceeded that for self-reported competence, consistent with the necessity-competence discrepancy discussed below. These descriptive patterns should be interpreted cautiously given the single-item dichotomous measurement approach.

### 3.3 Clustering-robust logistic regression results on 16 BCTs

**Figure 1:**
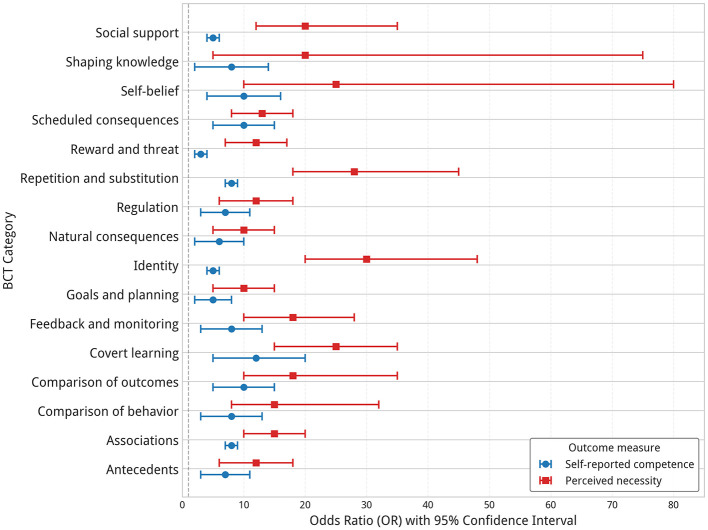
The clustering-robust logistic regression results. Lines illustrate 95% CIs, and the dots are OR values.

Figure 1 presents the logistic regression results; the full results are shown in Supplementary Table S5. After adjusting for confounders and accounting for district-level clustering via robust standard errors (with the caveat that only three district-level clusters limit the reliability of cluster-adjusted inference), perceived necessity and self-reported competence were positively associated with perceived usefulness for all 16 BCTs. All associations remained statistically significant after Benjamini-Hochberg false discovery rate (FDR) correction for multiple testing (FDR-adjusted *P* < 0.001).

For perceived necessity, the odds ratios (ORs) ranged from 8.91 (95% CI: 5.1–15.57) for the BCT “goals and planning” to 31.97 (95% CI: 21.23–48.13) for “identity.” For self-reported competence, ORs ranged from 4.08 (95% CI: 3.91–4.26) for “identity” to 14.04 (95% CI: 12.4–15.89) for “scheduled consequences.” As noted in the Methods section, the use of single-item dichotomous measures for all three constructs may have led to scale compression effects, which can mechanically inflate OR magnitudes in logistic regression. Thus, these ORs should not be interpreted as reflecting “strong substantive effects” in a clinical or practical sense, but rather as statistical indicators of the direction and consistency of associations within the constraints of the measurement strategy. In addition, the conceptual proximity between perceived necessity and perceived usefulness—both reflecting GPs' positive orientation toward a given BCT—may partly explain the large OR magnitudes for necessity. These associations should therefore not be interpreted as evidence of large practical or clinical effects, but rather as exploratory indicators of consistent positive co-occurrence patterns.

From a descriptive perspective, the OR for perceived necessity was numerically higher than that for self-reported competence in 15 of the 16 BCTs, with the only exception being “scheduled consequences” (competence OR = 14.04, 95% CI: 12.40–15.89; necessity OR = 13.68, 95% CI: 8.29–22.59). However, this observed numerical pattern does not constitute evidence of a statistically significant difference in the strength of association between the two constructs. Notably, ORs derived from separate logistic regression models (one per BCT) cannot be directly compared for statistical significance without constructing a unified modeling framework. Such a comparison was not the focus of this exploratory study, and the observed numerical pattern is presented here solely as a descriptive observation, not as an inferential conclusion.

Sensitivity analysis demonstrated consistent results between the original logistic regression and cluster-robust models, as detailed in Supplementary Table S2. District-stratified descriptive comparisons of endorsement rates showed broadly sup1 patterns across Changning (*n* = 192), Huangpu (*n* = 198), and Jinshan (*n* = 150) (Supplementary Table S3). Perceived necessity exceeded self-reported competence for all 16 BCTs in each district, with the largest necessity-competence gaps observed for Social support (Changning: 18.2 percentage points; Huangpu: 19.2; Jinshan: 20.0), Associations (15.1, 15.2, 12.7), and Feedback and monitoring (12.0, 17.2, 11.3). While Jinshan showed numerically higher endorsement rates overall (mean necessity 84.6% vs. 76.6% in Changning and 78.3% in Huangpu), the directional pattern—necessity consistently higher than competence—was uniform across districts. These descriptive sensitivity checks support the robustness of the observed association directions but do not overcome the limitations of cluster-robust inference with only three clusters or the absence of a unified model accounting for within-GP correlation.

## 4 Discussion

### 4.1 Principal findings

This cross-sectional study conducted in selected districts of Shanghai reveals that the majority of GPs acknowledge the significance of BCTs in managing older adults' health, while simultaneously perceiving digital technologies as potentially valuable tools for implementing these techniques. The analysis further indicates positive concurrent associations between both perceived necessity and self-reported competence with GPs' perceived usefulness of digital technologies in implementing BCTs. Descriptively, the numerical value of ORs for perceived necessity was higher than that for self-reported competence in most BCTs, but this observed numerical pattern is presented solely as a descriptive observation, not as an inferential conclusion regarding differential association strength. These observations collectively highlight a notable discrepancy between GPs' perception of the necessity of BCTs and their self-reported competence in implementing them.

GPs' perceptions regarding digital technology-supported BCTs showed descriptive variation across BCT types. Perceived necessity endorsement rates were consistently high across BCTs (72.0%−88.0%), whereas self-reported competence showed a somewhat wider descriptive spread (57.6%−80.4%). The numerical ranges of ORs from separate per-BCT models (necessity: 8.91–31.97; competence: 4.08–14.04) are presented strictly as descriptive patterns and should not be interpreted as formal tests of between-technique differences. Descriptively, larger necessity–competence gaps and relatively lower competence endorsement were observed for some less conventional BCT domains (e.g., Associations, social support), which may suggest that digital technologies could eventually assist GPs in these areas, but such interpretations remain exploratory. The findings contribute to our understanding of technology acceptance among healthcare professionals in selected Shanghai community health service settings.

From a theoretical standpoint, the positive associations observed across all 16 BCTs provide exploratory, hypothesis-generating evidence that patterns may be consistent with TAM and expectancy-value theory in selected Shanghai primary care settings. The consistent pattern—that both necessity and competence correlate with perceived usefulness—is consistent with these frameworks but does not confirm them, as core constructs (e.g., perceived ease of use, behavioral intention, subjective norms, autonomy) were not measured and the cross-sectional design precludes temporal ordering. Longitudinal research is needed to determine whether these cognitive indicators temporally precede actual technology adoption behavior. These findings are hypothesis-generating rather than confirmatory.

### 4.2 Comparison with prior work

The study found that GPs' perceived necessity of BCTs is higher than their competence across most assessed techniques. This observation aligns with prior research indicating that healthcare providers often recognize the value of behavior change interventions while reporting gaps in their practical skills to implement them. Digital technologies have been proposed as a potential means to mitigate such discrepancies, as supported by studies on medication adherence apps (24) and physical activity apps (44), which highlight how technology can lower implementation barriers and enhance the delivery of behavior change strategies.

Centered on perceived usefulness—a core construct in the Technology Acceptance Model (TAM)—this study identifies perceived necessity and self-reported competence as two key correlates of GPs' positive perceptions of digital technology-supported BCTs. Perceived necessity reflects an individual's recognition of the relevance of a technique to clinical practice, while self-reported competence captures self-assessed ability to implement it. This alignment with TAM's focus on cognitive antecedents of technology acceptance is consistent with broader healthcare informatics research, which emphasizes that providers' perceptions of need and capability are closely linked to their openness to adopting digital tools.

Notably, GPs' perceptions of necessity and competence descriptively varied across BCT types. This might be because healthcare providers' engagement with behavior change strategies is shaped by the inherent characteristics of the techniques themselves. For instance, techniques that align more closely with traditional primary care roles may be perceived as more familiar or feasible, while those involving less conventional components may elicit descriptively different patterns in perceived necessity and competence. Such variability underscores the importance of considering technique-specific perceptions when exploring digital technology integration in primary care, though no formal tests of differential associations were conducted in this study.

In the Chinese primary healthcare context, GPs, particularly referred to as “family doctors” in specific administrative regions such as Shanghai, fulfill a dual role as health gatekeepers and service providers through contract-based healthcare delivery systems. This service delivery model represents an essential component of China's basic public health services framework (45). Prior research in collectivist cultural settings has noted that normative influences and professional role expectations can influence providers' acceptance of new practices, which may help explain the observed variability in perceived necessity across BCT types (46). However, since constructs like subjective norms or behavioral intentions were not measured in this study, these interpretations remain exploratory and require further validation.

While the current study focuses on perceptual correlates, prior work has highlighted that external factors, such as time constraints (47), system-level support, and user-centered design (48, 49), also play critical roles in digital technology adoption. Therefore, future studies should build on this foundation by examining how these contextual factors interact with GPs' perceptions of necessity and competence to influence actual implementation behavior, as well as exploring technique-specific barriers to digital integration.

### 4.3 Strengths and limitations

To the best of our knowledge, this study represents the first survey in China to explore Chinese GPs' perceptions of using digital technologies to implement behavior change techniques (BCTs) for community-dwelling older adults, and the first to systematically examine the associations between perceived necessity, self-reported competence, and perceived usefulness a comprehensive set of 16 BCTs. These unique aspects enhance the study's exploratory significance and provide preliminary insights to inform future research and practice in China's aging context. The study sample comprised GPs from Changning, Huangpu, and Jinshan districts, which were strategically selected to reflect both urban and suburban areas, as well as diverse working patterns. Participants were recruited through convenience-based network sampling, which targeted all GPs within these districts.

The study has some limitations. First, this cross-sectional design precluded inference on causality. Second, the study was conducted in Shanghai, one of China's most economically developed and digitally advanced municipalities. GPs in Shanghai typically have higher educational attainment, greater exposure to digital health tools, and better infrastructure support than their counterparts in less-developed regions or rural China. Consequently, the generally positive perceptions of digital technology observed in this sample may not be generalizable to GPs in lower-tier cities or rural areas where digital literacy and resource availability are more limited. The three selected districts (Changning, Huangpu, and Jinshan) encompass both urban core and suburban settings, offering some internal diversity, but still within the context of a first-tier city. Third, non-response bias may affect the findings. GPs who are less digitally engaged or face greater time constraints may have opted out, potentially inflating the observed positive perceptions. Future surveys should employ follow-up procedures with non-respondents where ethically permissible. Fourth, the measurement strategy has inherent limitations. Given the need to assess 16 BCTs, we adopted single-item dichotomous measures to reduce GP response burden. However, these measures serve only as coarse proxies for the complex cognitive constructs. Dichotomization compresses variable variability, leading to information loss and potential misclassification bias, and may mechanically inflate ORs in logistic regression. While several authors have defended the psychometric properties of single-item scales (50, 51), single-response measures can therefore be used for perceptions [e.g., necessity (36)]. Still, we encourage future studies to use more sensitive and specific measures. Fifth, participants were recruited through convenience-based professional networks within three districts. Even within Shanghai, this approach may overrepresent digitally connected or professionally engaged GPs and underrepresent clinicians with limited access to survey channels. Sixth, district-level cluster-robust inference was applied with only three clusters (Changning, Huangpu, and Jinshan), which limits the reliability of cluster-adjusted standard errors; inferential results from these models should be interpreted as exploratory rather than definitive. Seventh, each GP provided responses for 16 BCTs, creating within-respondent dependence that district-level cluster-robust models and separate per-BCT regressions do not fully address. We explored a unified mixed-effects logistic regression with GP-level random intercepts and BCT fixed effects on long-format data, but this specification did not yield stable parameter estimates in our analysis pipeline (see Methods); we were therefore unable to obtain reliable unified-model inference for within-GP correlation. Descriptive comparisons of patterns across BCTs should therefore not be interpreted as if based on independent observations. Finally, the conceptual proximity between perceived necessity and perceived usefulness may partly inflate observed associations, in addition to limitations arising from dichotomous single-item measurement and scale compression.

### 4.4 Implications

This study lays a foundation for future research to explore GPs' perceptions of digital technology-supported behavior change interventions for community-dwelling older adults. Future research should adopt multi-item, continuous measures for perceived necessity, self-reported competence, and perceived usefulness to reduce information loss and scale compression; use unified mixed-effects models to account for within-GP and within-district dependence; and use longitudinal study designs to examine the temporal relationships between these cognitive constructs and actual digital technology adoption behavior. Preliminarily, the observed discrepancy between GPs' perceived necessity and self-reported competence suggests that future capacity-building efforts should address this gap.

Based on the descriptive necessity-competence gap, we offer preliminary, feasibility-oriented implications for practice—requiring validation in broader samples:

GP training: Targeted BCT skill-building should prioritize techniques with among the largest descriptively observed necessity-competence gaps in this sample, such as Associations (57.6% competence vs. 72.0% necessity), Comparison of outcomes (60.9% vs. 73.9%), Scheduled consequences (59.8% vs. 73.0%), and Social support (63.7% vs. 82.8%). Brief, workflow-integrated training modules embedded in existing family doctor contract service programs may be more feasible than standalone workshops given GPs' time constraints.

Digital tool design: Digital supports may be most useful for scaffolding less familiar BCT domains with larger descriptively observed necessity–competence gaps in this sample (e.g., cueing/associations and social support coordination), rather than focusing only on information-delivery techniques (e.g., shaping knowledge), where self-reported competence was already relatively higher (80.4%).

Workflow support: Embedding brief BCT prompts and digital decision-support within consultation templates and chronic disease management workflows could help GPs deliver BCTs within limited consultation time.

Implementation strategy: Rollout of digital BCT tools may be most impactful for BCT groups with both high perceived necessity and relatively lower self-reported competence, where digital scaffolding could bridge the identified gap.

For healthcare policy, this exploratory study identified a perceived need among GPs to utilize BCTs for older adults' health management, with digital technologies emerging as a potential facilitator. These preliminary observations warrant cautious interpretation and require validation through more robust methodologies before informing policy development. Further research is needed to substantiate whether targeted investments in digital health tools or professional development align with GPs' actual practice needs and improve care outcomes.

## 5 Conclusions

This exploratory study provides initial insights into GPs' perceptions in selected Shanghai community health service settings regarding the use of digital technologies to implement BCTs for community-dwelling older adults. The findings reveal positive associations between perceived necessity, self-reported competence, and perceived usefulness of digital technology-supported BCTs, as well as a notable discrepancy between GPs' recognition of BCT necessity and their self-reported competence in implementing these techniques. Digital technologies may offer potential opportunities to support GPs in delivering BCTs, but claims regarding differential benefits for specific BCTs are not supported by formal statistical tests. These tentative findings highlight the need for future research to validate the observed patterns using more rigorous methods including multi-item measures and longitudinal designs. Therefore, the findings serve as a starting point for hypothesis generation, emphasizing the value of further research to inform evidence-based capacity-building initiatives and digital health strategies in primary care geriatric management.

## Data Availability

The raw data supporting the conclusions of this article will be made available by the authors, without undue reservation.
