# Supplementary material for: GPs' perceptions of digital technology for behavior change interventions for community-dwelling older adults: a cross-sectional study
Source: Front Public Health. 2026 Jul 15;14:1849754. doi: 10.3389/fpubh.2026.1849754 (PMC13416695; doi:10.3389/fpubh.2026.1849754)
Supplement: Supplementary file 1 [file Supplementary_file_1.pdf]

GPs' perceptions of digital technology for behavior change interventions for community-dwelling older adults: a cross-sectional study

Supplementary file

Supplementary Table S1. Examples of 16 BCTs

Supplementary A. The Questionnaire-General Practitioners' Perceptions of BCTs

Supplementary Table S2. Results of the original logistic regression and clustering-robust logistic regression

Supplementary Table S3. District-stratified endorsement rates for perceived necessity, self-reported competence, and perceived usefulness across 16 BCT groups

Supplementary Table S4. Endorsement rates for perceived necessity, self-reported competence, and perceived usefulness of digital technology-supported BCTs across 16 groups (N=540)

Supplementary Table S5. Results of Clustering-Robust Logistic Regression

Supplementary Table S1. Examples of 16 BCTs

Full Name	Examples
Goals and planning	Goal setting (behavior), Problem solving, Goal setting (outcome), Action planning, Review behavior goal(s), Discrepancy between current behavior and goal, Review outcome goal(s), Behavioral contract, Commitment.
Feedback and monitoring	Monitoring of behavior by others without feedback, Feedback on behavior, Self-monitoring of behavior, Self-monitoring of outcome(s) of behavior, Monitoring of outcome(s) of behavior without feedback, Biofeedback, Feedback on outcome(s) of behavior
Social Support	Social support (unspecified), Social support (practical), Social support (emotional)
Shaping Knowledge	Instruction on how to perform the behavior, Information about Antecedents, Re-attribution, and Behavioral experiments
Natural Consequences	Information about health consequences, Salience of

	consequences, Information about social and environmental consequences, Monitoring of emotional consequences, Anticipated regret, Information about emotional consequences
Comparison of Behavior	Demonstration of the behavior, Social comparison, Information about others' approval
Associations	Prompts/cues, Cue signalling reward, Reduce prompts/cues, Remove access to the reward, Remove aversive stimulus, Satiation, Exposure, Associative learning.
Repetition and Substitution	Behavioral practice/rehearsal, Behavior substitution, Habit formation, Habit reversal, Overcorrection, Generalisation of target behavior, Graded tasks
Covert Learning	Imaginary punishment, Imaginary reward, Vicarious consequences
Reward and Threat	Material incentive (behavior), Material reward (behavior), Non-specific reward, Social reward, Social incentive, Non-specific incentive, Self-incentive, Incentive (outcome), Self-reward, Reward (outcome), Future punishment
Regulation	Pharmacological support, reducing negative emotions, conserving mental resources, and Paradoxical instructions.
Antecedents	Restructuring the physical environment, Restructuring the social environment, Avoidance/reducing exposure to cues for the behavior, Distraction, Adding objects to the environment, Body changes
Identity	Identification of self as role model, Framing/reframing, Incompatible beliefs, Valued self-identity, Identity associated with changed behavior
Scheduled Consequences	Behavior cost, Punishment, Remove reward, Reward approximation, Rewarding completion, Situation-specific reward, Reward incompatible behavior, Reward alternative behavior, Reduce reward frequency, Remove punishment.
Self-belief	Verbal persuasion about capability, Mental rehearsal of successful performance, Focus on past success, Self-talk
Comparison of Outcomes	Credible source, Pros and cons, Comparative imagining of future outcomes

Supplementary A-The Questionnaire-General Practitioners' Perceptions of BCTs

Q1: What is your gender? [Single Choice]

A. Male

B. Female

Q2: What is your age group? [Single Choice]

A. Under 30 years

B. 30-39 years

C. 40-49 years

D. 50 years and above

Q3: What is your highest educational qualification? [Single Choice]

A. Associate degree and below

B. Bachelor's degree

C. Master's degree and above

Q4: What is your professional title? [Single Choice]

Intermediate title and below

Senior title

Q5: How many years have you been working? [Single Choice]

0-5

6-10

11-15

16-20

>20

Q6: In the context of the "Initiative for Active Health and Technological Responses to Population Aging," the following are some health behavior change techniques (BCTs). Please indicate your perceptions regarding the use of these tools for optimizing health behaviors and improving health management among older adults.

BCT Item	Please rate based on your perceptions toward the necessity of the BCT in your practice. (Yes, or No)	Do you think you have the ability to conduct the BCT? (Yes, or No)	Do you think digital technologies (e.g., ICT, decision support systems) can assist you in conducting the BCT (e.g., improve your intervention, make your intervention more effective or easier)? (Yes, or No)
1. Goals and planning - Set or agree on a goal defined in terms of the behavior to be achieved.			

2. Feedback and monitoring - Monitor and provide informative or evaluative feedback on the performance of the behavior.			
3. Social support - Advise on/arrange social support (e.g., from friends, relatives, colleagues, 'buddies' or staff) or non-contingent praise or reward for performance of the behavior.			
4. Shaping Knowledge - Advise or agree on how to perform the behavior.			
5. Natural Consequences - Provide information (e.g., written, verbal, visual) about the health consequences of performing the behavior.			
6. Comparison of Behaviour - Provide an observable sample of the performance of the behavior, directly in person or indirectly, e.g., via video, picture, or audio.			
7. Associations - Introduce or define environmental or social stimulus to prompt or cue the behavior.			
8. Repetition and Substitution - Prompt practice or rehearsal of the performance of the behavior one or more times in a context or at a time when the performance may not be necessary, to increase habit and skill.			
9. Covert Learning - Establish a method for the person to monitor and record their behavior(s) as part of a behavior change strategy.			
10. Reward and Threat - Inform that rewards will be delivered or aversive consequences removed after successful action.			
11. Regulation - Prompt rehearsal and repetition of the behavior in			

the same context repeatedly so that the context elicits the behavior.			
12. Antecedents - Advise or arrange changes to the physical environment.			
13. Identity - Advise the person to construct a new self-identity around the changed behavior or to note that the new behavior is consistent with an aspect of their identity.			
14. Scheduled consequences - Arrange for aversive or rewarding consequences to follow performance of the unwanted behavior.			
15. Self-belief - Tell the person that they can successfully perform the wanted behavior, arguing against self-doubts and asserting that they can and will succeed.			
16. Comparison of Outcomes - Prompt thinking about and/or visualization of the (un)desirable outcomes/experiences of performing/not performing the behavior.			

Supplementary Table S2. Results of the original logistic regression and clustering-robust logistic regression

		the original logistic regression	clustering-robust logistic regression
		Odds ratio (95%CI)	Odds ratio (95%CI)
Goals and planning	necessity	8.9*** (4.7 – 16.8)	8.91***(5.1-15.57)
	competence	4.4*** (2.5 – 7.6)	4.38***(2.92-6.56)
Feedback and monitoring	necessity	19.5*** (10.4 – 36.5)	18.89***(10.71-33.3)
	competence	5.9*** (3.3 – 10.4)	5.86***(2.82-12.19)
Social support	necessity	20.5*** (9.9 – 42.6)	20.49***(11.67-35.99)
	competence	4.2*** (2.5 – 6.9)	4.2***(4.01-4.39)
Shaping knowledge	necessity	20.0*** (8.7 – 45.9)	19.45***(5-75.75)
	competence	5.7*** (3.1 – 10.5)	5.79***(2.37-14.13)
Natural consequences	necessity	9.4*** (4.6 – 19.3)	9.21***(5.43-15.62)
	competence	4.8*** (2.7 – 8.7)	4.65***(2.75-7.87)
Comparison of behavior	necessity	13.1*** (6.9 – 25.1)	13.1***(5.01-34.25)
	competence	5.3*** (3.0 – 9.5)	5.3***(2.05-13.72)
Associations	necessity	15.5*** (8.1 – 29.4)	15.08***(11.32-20.08)
	competence	7.5 ***(4.1 – 13.7)	7.41***(6.45-8.5)
Repetition and substitution	necessity	28.3*** (13.2 – 61.0)	27.79***(17.54-44.03)
	competence	7.5 ***(4.2 – 13.3)	7.23***(6.61-7.9)
Covert learning	necessity	27.9*** (12.8 – 60.9)	26.6***(18.89-37.47)
	competence	7.6*** (4.2 – 13.6)	7.43***(2.51-21.96)
Reward and threat	necessity	12.3*** (6.3 – 23.8)	12.33***(8.18-18.59)
	competence	4.3*** (2.5 – 7.5)	4.3***(3.77-4.9)
Regulation	necessity	10.5*** (4.7 – 23.4)	10.71***(5.24-21.88)
	competence	7.6*** (4.2 – 13.9)	7.56***(4.61-12.39)
Antecedent	necessity	11.9 ***(6.1 – 23.3)	11.78***(6.92-20.04)
	competence	7.7*** (4.5 – 13.3)	7.69***(5.71-10.36)
Identity	necessity	31.6*** (14.0 – 71.2)	31.97***(21.23-48.13)
	competence	4.1*** (2.2 – 7.5)	4.08***(3.91-4.26)
Scheduled consequences	necessity	13.7 ***(6.8 – 27.4)	13.68***(8.29-22.59)
	competence	14.1*** (7.6 – 26.2)	14.04***(12.4-15.89)
Self-belief	necessity	25.0*** (10.1 – 62.2)	25.05***(8.02-78.2)
	competence	8.5*** (4.5 – 16.2)	8.42***(4.62-15.36)
Comparison of outcomes	necessity	18.7*** (8.8 – 39.6)	18.52***(9.64-35.55)
	competence	8.6*** (4.7 – 15.8)	8.58***(5.5-13.38)

Supplementary Table S3. District-stratified endorsement rates for perceived necessity, self-reported competence, and perceived usefulness across 16 BCT groups

BCT	Metric	Changning (n=192)	Huangpu (n=198)	Jinshan (n=150)
Goals and planning	Necessity	79.2	83.8	90.0
	Competence	70.3	68.2	75.3
	Usefulness	75.5	80.8	84.7
Feedback and monitoring	Necessity	75.0	78.8	84.7
	Competence	63.0	61.6	73.3
	Usefulness	71.9	76.3	80.0
Social support	Necessity	77.1	82.8	90.0
	Competence	58.9	63.6	70.0
	Usefulness	64.1	72.2	80.0
Shaping knowledge	Necessity	85.9	85.9	93.3
	Competence	81.2	75.8	85.3
	Usefulness	77.1	77.8	83.3
Natural consequences	Necessity	81.8	84.8	92.7
	Competence	76.0	76.3	82.7
	Usefulness	74.0	76.3	80.7
Comparison of behavior	Necessity	75.0	79.3	84.7
	Competence	69.8	67.7	80.0
	Usefulness	70.3	75.3	78.0
Associations	Necessity	69.3	69.7	78.7
	Competence	54.2	54.5	66.0
	Usefulness	67.7	66.7	72.7
Repetition and substitution	Necessity	74.5	75.3	81.3
	Competence	59.4	61.1	66.7
	Usefulness	67.2	65.2	71.3
Covert learning	Necessity	73.4	71.7	77.3
	Competence	58.3	60.6	64.7
	Usefulness	62.5	63.6	69.3
Reward and threat	Necessity	75.0	75.3	82.7
	Competence	69.8	64.6	73.3
	Usefulness	66.1	67.7	68.7
Regulation	Necessity	82.8	82.8	89.3
	Competence	76.6	71.2	80.7
	Usefulness	71.9	73.2	79.3
Antecedents	Necessity	76.0	76.8	80.7
	Competence	63.0	64.6	70.0
	Usefulness	66.1	67.7	74.0
Identity	Necessity	76.0	79.3	82.0

	Competence	69.8	68.2	75.3
	Usefulness	67.2	69.7	72.0
Scheduled consequences	Necessity	69.3	72.2	78.7
	Competence	62.0	57.1	60.7
	Usefulness	67.2	64.1	68.7
Self-belief	Necessity	78.6	79.3	85.3
	Competence	72.4	71.2	78.0
	Usefulness	68.8	70.2	77.3
Comparison of outcomes	Necessity	76.0	75.8	82.0
	Competence	69.8	67.2	70.0
	Usefulness	70.8	70.2	71.3

Note: All values are percentages. In all three districts, perceived necessity exceeded self-reported competence for all 16 BCT groups. This table is descriptive only and does not constitute formal inference about between-district differences.

Supplementary Table S4. Endorsement rates for perceived necessity, self-reported competence, and perceived usefulness of digital technology-supported BCTs across 16 groups (N=540)

BCT	Necessity n (%)	Competence n (%)	Usefulness n (%)
Goals and planning	453 (83.9)	383 (70.9)	432 (80.0)
Feedback and monitoring	427 (79.1)	353 (65.4)	409 (75.7)
Social support	447 (82.8)	344 (63.7)	386 (71.5)
Shaping knowledge	475 (88.0)	434 (80.4)	427 (79.1)
Natural consequences	464 (85.9)	421 (78.0)	414 (76.7)
Comparison of behavior	428 (79.3)	388 (71.9)	401 (74.3)
Associations	389 (72.0)	311 (57.6)	371 (68.7)
Repetition and substitution	414 (76.7)	335 (62.0)	365 (67.6)
Comparison of outcomes	399 (73.9)	329 (60.9)	350 (64.8)
Reward and threat	417 (77.2)	372 (68.9)	364 (67.4)
Regulation	457 (84.6)	409 (75.7)	402 (74.4)
Antecedents	419 (77.6)	354 (65.6)	372 (68.9)
Identity	426 (78.9)	382 (70.7)	375 (69.4)
Scheduled consequences	394 (73.0)	323 (59.8)	359 (66.5)
Self-belief	436 (80.7)	397 (73.5)	387 (71.7)
Covert learning	419 (77.6)	372 (68.9)	382 (70.7)

Supplementary Table S5. Results of Clustering-Robust Logistic Regression

BCTs	Variables	Odds ratio（95%CI）
Goals and planning	Necessity	8.91***(5.1-15.57)
	Competence	4.38***(2.92-6.56)
Feedback and monitoring	Necessity	18.89***(10.71-33.3)
	Competence	5.86***(2.82-12.19)
Social support	Necessity	20.49***(11.67-35.99)
	Competence	4.2***(4.01-4.39)
Shaping knowledge	Necessity	19.45***(5-75.75)
	Competence	5.79***(2.37-14.13)
Natural consequences	Necessity	9.21***(5.43-15.62)
	Competence	4.65***(2.75-7.87)
Comparison of behavior	Necessity	13.1***(5.01-34.25)
	Competence	5.3***(2.05-13.72)
Associations	Necessity	15.08***(11.32-20.08)
	Competence	7.41***(6.45-8.5)
Repetition and substitution	Necessity	27.79***(17.54-44.03)
	Competence	7.23***(6.61-7.9)
Covert learning	Necessity	26.6***(18.89-37.47)
	Competence	7.43***(2.51-21.96)
Reward and threat	Necessity	12.33***(8.18-18.59)
	Competence	4.3***(3.77-4.9)
Regulation	Necessity	10.71***(5.24-21.88)
	Competence	7.56***(4.61-12.39)
Antecedents	Necessity	11.78***(6.92-20.04)
	Competence	7.69***(5.71-10.36)
Identity	Necessity	31.97***(21.23-48.13)
	Competence	4.08***(3.91-4.26)
Scheduled consequences	Necessity	13.68***(8.29-22.59)
	Competence	14.04***(12.4-15.89)

Self-belief	Necessity	25.05***(8.02-78.2)
	Competence	8.42***(4.62-15.36)
Comparison of outcomes	Necessity	18.52***(9.64-35.55)
	Competence	8.58***(5.5-13.38)

Note: we conducted 16 clustering-robust logistic regression models. For each BCT, perceived usefulness served as the dependent variable, with perceived necessity and self-reported competence as core independent variables. Sociodemographic characteristics (sex, age, educational background, professional title, and years of work) were included as confounders.

***: $P<0.001$
